# 4-Bromo-3-{*N*-[2-(3,4-dimethoxy­phen­yl)eth­yl]-*N*-methyl­sulfamo­yl}-5-methyl­benzoic acid monohydrate

**DOI:** 10.1107/S1600536809022041

**Published:** 2009-06-13

**Authors:** C. Suneel Manohar Babu, Helen P. Kavitha, Jasmine P. Vennila, G. Chakkaravarthi, V. Manivannan

**Affiliations:** a Nicholas Piramal Research Centre, Nicholas Piramal India Limited, Mumbai 400 063, India; b Department of Chemistry, SRM University, Ramapuram, Chennai 600 089, India; c Department of Physics, Panimalar Institute of Technology, Chennai 600 095, India; d Department of Physics, CPCL Polytechnic College, Chennai 600 068, India; e Department of Research and Development, PRIST University, Vallam, Thanjavur 613 403, Tamil Nadu, India

## Abstract

In the title compound, C_19_H_22_BrNO_6_S·H_2_O, the dihedral angle between the planes of the two benzene rings is 3.1 (1)°. These rings are stacked over one another with their centroids separated by 3.769 (2) Å, indicating weak π–π inter­actions. In the crystal structure, mol­ecules are linked by O—H⋯O and O—H⋯(O,O) hydrogen bonds involving the water mol­ecule, forming a two-dimensional network parallel to (001).

## Related literature

For the biological activity of sulfonamides, see: Cates (1986 ▶); Steele & Beran (1984 ▶); Benedetti (1987 ▶); Mengelers *et al.* (1997 ▶). For related structures, see: Babu *et al.* (2009*a* ▶,*b* ▶); Shad *et al.* (2009 ▶); For graph-set notation, see: Bernstein *et al.* (1995 ▶)
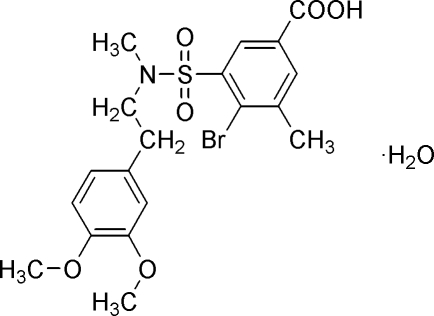


## Experimental

### 

#### Crystal data

C_19_H_22_BrNO_6_S·H_2_O
*M* *_r_* = 490.36
Orthorhombic, 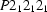

*a* = 7.7938 (2) Å
*b* = 7.8280 (2) Å
*c* = 34.6549 (8) Å
*V* = 2114.29 (9) Å^3^
*Z* = 4
Mo *K*α radiation
μ = 2.08 mm^−1^
*T* = 295 K
0.15 × 0.12 × 0.10 mm

#### Data collection

Bruker Kappa APEXII area-detector diffractometer
Absorption correction: multi-scan (*SADABS*; Sheldrick, 1996 ▶) *T*_min_ = 0.745, *T*_max_ = 0.819
12583 measured reflections
4619 independent reflections
3427 reflections with *I* > 2σ(*I*)
*R*_int_ = 0.028

#### Refinement

*R*[*F* ^2^ > 2σ(*F* ^2^)] = 0.040
*wR*(*F* ^2^) = 0.107
*S* = 1.05
4619 reflections
275 parameters
3 restraints
H atoms treated by a mixture of independent and constrained refinement
Δρ_max_ = 0.36 e Å^−3^
Δρ_min_ = −0.30 e Å^−3^
Absolute structure: Flack (1983 ▶), 1915 Friedel pairs
Flack parameter: −0.004 (10)

### 

Data collection: *APEX2* (Bruker, 2004 ▶); cell refinement: *SAINT* (Bruker, 2004 ▶); data reduction: *SAINT*; program(s) used to solve structure: *SHELXS97* (Sheldrick, 2008 ▶); program(s) used to refine structure: *SHELXL97* (Sheldrick, 2008 ▶); molecular graphics: *PLATON* (Spek, 2009 ▶); software used to prepare material for publication: *SHELXL97*.

## Supplementary Material

Crystal structure: contains datablocks global, I. DOI: 10.1107/S1600536809022041/ci2818sup1.cif

Structure factors: contains datablocks I. DOI: 10.1107/S1600536809022041/ci2818Isup2.hkl

Additional supplementary materials:  crystallographic information; 3D view; checkCIF report

## Figures and Tables

**Table 1:** Hydrogen-bond geometry (Å, °)

*D*—H⋯*A*	*D*—H	H⋯*A*	*D*⋯*A*	*D*—H⋯*A*
O2—H2*A*⋯O1*W*	0.82	1.73	2.535 (4)	169
O1*W*—H1*W*⋯O1^i^	0.79 (3)	1.97 (4)	2.732 (5)	162 (6)
O1*W*—H2*W*⋯O5^ii^	0.75 (3)	2.46 (4)	3.066 (5)	139 (5)
O1*W*—H2*W*⋯O6^ii^	0.75 (3)	2.14 (4)	2.828 (5)	154 (5)

Symmetry codes: (i) 
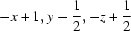
; (ii) 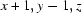.

**Fig. 1.**
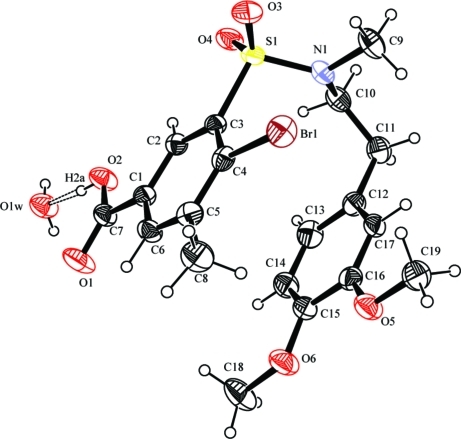
The molecular structure of the title compound, with atom labels and 30% probability displacement ellipsoids for non-H atoms. The dashed line denotes a hydrogen bond.

## supplementary crystallographic information

### Comment

Sulfonamides are a class of anti-microbial agents that have seen extensive use in medicine. They are the first agents to be used for the treatment of bacterial infection (Cates, 1986). Sulfonamides are used to treat atrophic rhinitis in swine and heart water in cattle and many other diseases in a variety of animals (Steele & Beran, 1984). In adition sulfonamides have a variety of biological activities such as antibacterial, antimalarial and antileprotic agents (Benedetti, 1987; Mengelers *et al.*, 1997).

The geometric parameters of the title molecule agree well with those reported for similar structures (Babu *et al.*, 2009*a*,*b*; Shad *et al.*, 2009). In the molecular structure, the two benzene rings are stacked over one another with their centroids separated by 3.769 (2) Å, indicating a weak π-π interaction. The dihedral angle between the two benzene rings is 3.1 (1)°. A distorted tetrahedral geometry [O3—S1—O4 = 118.7 (2)° and O3—S1—N1 = 108.8 (2) °] is observed around S1 atom.

In the crystal structure, O—H···O hydrogen bonds (Table 1) link the molecules into a two-dimensional network parallel to the (0 0 1). The O1W—H2W···O5 and O1W—H2W···O6 bifurcated donar bonds generate an *R*_1_^2^(5) ring motif (Bernstein *et al.*, 1995).

### Experimental

A solution of 2-(3,4-dimethoxyphenyl)-*N*-methyl ethanamine (1 g, 0.0051 mol) in ethyl acetate (20 ml) was charged into a round bottom flask equipped with thermometer pocket, condenser and guard tube. Then, pyridine (0.81 g, 0.0102 mol) was added at 25–30°C. After 5 min stirring, 2-bromo-5-(chlorosulfonyl)-3-methylbenzoic acid (0.96 g, 0.00307 mol) was charged into the reaction mass and heated to 45-50°C and maintained for 5-6 h. The completion of the reaction was checked by thin layer chromatography (1:1 hexane-ethyl acetate) and the reaction mass was cooled to 25–30 °C and quenched with 20 ml of water. Then, the aqueous layer was separated and the ethyl acetate layer was washed twice with 10% sodium chloride solution and dried over 2 g of anhydrous sodium sulfate. The solvent was distilled under vacuum at 35-40°C and the crude compound isolated. The crude compound was purified through column chromatography using hexane and ethyl acetate as eluents.

### Refinement

Atoms H1W and H2W were located in a difference difference Fourier map and refined freely. Other H atoms were positioned geometrically and refined using a riding model with C-H = 0.93 Å and *U*_iso_(H) = 1.2U_eq_(C) for aromatic, C-H = 0.97 Å and *U*_iso_(H) = 1.5U_eq_(C) for methylene, C-H = 0.96 Å and *U*_iso_(H) = 1.5U_eq_(C) for methyl and O-H = 0.82 Å and *U*_iso_(H) = 1.2U_eq_(O) for water H atoms. The O1W-H1W and O1W-H2W distances were restrained to 0.82 (4) Å and atoms O2 and C7 were subjected to a rigid bond restraint.

### Crystal data

**Table:**

C_19_H_22_BrNO_6_S·H_2_O	*F*(000) = 1008
*M**_r_* = 490.36	*D*_x_ = 1.540 Mg m^−^^3^
Orthorhombic, *P*2_1_2_1_2_1_	Mo *K*α radiation, λ = 0.71073 Å
Hall symbol: P 2ac 2ab	Cell parameters from 12583 reflections
*a* = 7.7938 (2) Å	θ = 1.2–27.2°
*b* = 7.8280 (2) Å	µ = 2.08 mm^−^^1^
*c* = 34.6549 (8) Å	*T* = 295 K
*V* = 2114.29 (9) Å^3^	Block, colourless
*Z* = 4	0.15 × 0.12 × 0.10 mm

### Data collection

**Table:**

Bruker Kappa APEXII area-detector diffractometer	4619 independent reflections
Radiation source: fine-focus sealed tube	3427 reflections with *I* > 2σ(*I*)
graphite	*R*_int_ = 0.028
ω and φ scans	θ_max_ = 27.2°, θ_min_ = 1.2°
Absorption correction: multi-scan (*SADABS*; Sheldrick, 1996)	*h* = −10→6
*T*_min_ = 0.745, *T*_max_ = 0.819	*k* = −9→10
12583 measured reflections	*l* = −44→44

### Refinement

**Table:**

Refinement on *F*^2^	Secondary atom site location: difference Fourier map
Least-squares matrix: full	Hydrogen site location: inferred from neighbouring sites
*R*[*F*^2^ > 2σ(*F*^2^)] = 0.040	H atoms treated by a mixture of independent and constrained refinement
*wR*(*F*^2^) = 0.107	*w* = 1/[σ^2^(*F*_o_^2^) + (0.0512*P*)^2^] where *P* = (*F*_o_^2^ + 2*F*_c_^2^)/3
*S* = 1.05	(Δ/σ)_max_ = 0.001
4619 reflections	Δρ_max_ = 0.36 e Å^−^^3^
275 parameters	Δρ_min_ = −0.29 e Å^−^^3^
3 restraints	Absolute structure: Flack (1983), 1915 Friedel pairs
Primary atom site location: structure-invariant direct methods	Flack parameter: −0.004 (10)

### Fractional atomic coordinates and isotropic or equivalent isotropic displacement parameters (Å^2^)

**Table:**

	*x*	*y*	*z*	*U*_iso_*/*U*_eq_	
C1	0.4622 (4)	−0.0786 (5)	0.33480 (10)	0.0437 (9)	
C2	0.4708 (4)	−0.1063 (5)	0.37399 (10)	0.0409 (8)	
H2	0.5171	−0.2075	0.3835	0.049*	
C3	0.4107 (4)	0.0161 (4)	0.39913 (9)	0.0379 (8)	
C4	0.3406 (4)	0.1659 (5)	0.38498 (10)	0.0428 (8)	
C5	0.3306 (5)	0.1973 (5)	0.34527 (11)	0.0490 (9)	
C6	0.3940 (5)	0.0735 (5)	0.32070 (11)	0.0495 (9)	
H6	0.3910	0.0921	0.2942	0.059*	
C7	0.5272 (5)	−0.2101 (5)	0.30749 (10)	0.0500 (9)	
C8	0.2547 (7)	0.3601 (5)	0.32931 (12)	0.0714 (12)	
H8A	0.1351	0.3657	0.3358	0.107*	
H8B	0.2674	0.3615	0.3018	0.107*	
H8C	0.3133	0.4566	0.3402	0.107*	
C9	0.1792 (6)	0.0519 (6)	0.49724 (11)	0.0665 (12)	
H9A	0.0653	0.0908	0.4915	0.100*	
H9B	0.2535	0.1485	0.5007	0.100*	
H9C	0.1774	−0.0148	0.5205	0.100*	
C10	0.1537 (5)	−0.2158 (5)	0.45884 (11)	0.0529 (10)	
H10A	0.1564	−0.2827	0.4824	0.063*	
H10B	0.2153	−0.2791	0.4392	0.063*	
C11	−0.0297 (5)	−0.1940 (6)	0.44630 (12)	0.0606 (11)	
H11A	−0.0842	−0.3054	0.4459	0.073*	
H11B	−0.0889	−0.1251	0.4654	0.073*	
C12	−0.0524 (5)	−0.1125 (5)	0.40751 (11)	0.0511 (10)	
C13	−0.0043 (5)	−0.1982 (6)	0.37419 (13)	0.0625 (11)	
H13	0.0431	−0.3069	0.3762	0.075*	
C14	−0.0251 (5)	−0.1263 (6)	0.33830 (12)	0.0579 (11)	
H14	0.0064	−0.1866	0.3163	0.069*	
C15	−0.0922 (5)	0.0336 (6)	0.33519 (10)	0.0527 (10)	
C16	−0.1403 (4)	0.1245 (5)	0.36809 (11)	0.0484 (9)	
C17	−0.1194 (4)	0.0509 (5)	0.40371 (10)	0.0481 (9)	
H17	−0.1507	0.1116	0.4257	0.058*	
C18	−0.0805 (8)	0.0331 (7)	0.26626 (12)	0.0896 (16)	
H18A	0.0405	0.0099	0.2655	0.134*	
H18B	−0.1111	0.1043	0.2448	0.134*	
H18C	−0.1428	−0.0725	0.2648	0.134*	
C19	−0.2491 (6)	0.3849 (5)	0.39391 (12)	0.0633 (10)	
H19A	−0.3342	0.3264	0.4089	0.095*	
H19B	−0.2942	0.4926	0.3854	0.095*	
H19C	−0.1489	0.4042	0.4094	0.095*	
N1	0.2420 (5)	−0.0523 (4)	0.46561 (7)	0.0491 (7)	
O1	0.5144 (5)	−0.1988 (5)	0.27278 (7)	0.0826 (10)	
O2	0.5954 (4)	−0.3405 (4)	0.32450 (7)	0.0634 (7)	
H2A	0.6330	−0.4068	0.3082	0.095*	
O3	0.5148 (4)	0.1079 (4)	0.46804 (8)	0.0690 (8)	
O4	0.5175 (4)	−0.1965 (4)	0.45044 (7)	0.0609 (8)	
O5	−0.2047 (4)	0.2842 (4)	0.36146 (7)	0.0624 (8)	
O6	−0.1214 (4)	0.1172 (4)	0.30090 (7)	0.0683 (8)	
S1	0.43421 (12)	−0.03348 (13)	0.44937 (2)	0.0467 (2)	
Br1	0.25758 (6)	0.33631 (5)	0.418916 (12)	0.06382 (16)	
O1W	0.7092 (5)	−0.5755 (5)	0.28115 (11)	0.0720 (10)	
H2W	0.749 (6)	−0.644 (5)	0.2931 (13)	0.074 (18)*	
H1W	0.641 (6)	−0.627 (7)	0.2690 (14)	0.09 (2)*	

### Atomic displacement parameters (Å^2^)

**Table:**

	*U*^11^	*U*^22^	*U*^33^	*U*^12^	*U*^13^	*U*^23^
C1	0.0415 (18)	0.050 (2)	0.0399 (18)	−0.0069 (17)	−0.0028 (15)	−0.0013 (17)
C2	0.0412 (17)	0.040 (2)	0.0419 (18)	−0.0027 (15)	−0.0011 (15)	0.0009 (17)
C3	0.0372 (16)	0.042 (2)	0.0350 (16)	−0.0057 (15)	−0.0030 (14)	0.0002 (16)
C4	0.0365 (16)	0.0367 (19)	0.055 (2)	−0.0073 (16)	0.0003 (15)	−0.0056 (18)
C5	0.0495 (19)	0.042 (2)	0.056 (2)	−0.0081 (17)	−0.0055 (17)	0.0097 (19)
C6	0.057 (2)	0.054 (2)	0.0380 (18)	−0.0078 (19)	−0.0009 (16)	0.0117 (19)
C7	0.055 (2)	0.057 (2)	0.038 (2)	−0.0041 (16)	0.0011 (16)	−0.0068 (16)
C8	0.084 (3)	0.059 (3)	0.072 (3)	0.001 (3)	−0.001 (3)	0.021 (2)
C9	0.089 (3)	0.063 (3)	0.048 (2)	0.005 (2)	0.016 (2)	−0.013 (2)
C10	0.073 (3)	0.043 (2)	0.043 (2)	−0.0030 (19)	0.0064 (18)	0.0065 (17)
C11	0.061 (2)	0.064 (3)	0.057 (2)	−0.008 (2)	0.008 (2)	0.011 (2)
C12	0.0433 (18)	0.058 (3)	0.052 (2)	−0.0118 (18)	0.0007 (18)	−0.0030 (19)
C13	0.063 (2)	0.056 (3)	0.068 (3)	−0.003 (2)	0.003 (2)	−0.011 (2)
C14	0.063 (2)	0.060 (3)	0.050 (2)	0.000 (2)	0.0018 (19)	−0.015 (2)
C15	0.051 (2)	0.066 (3)	0.0408 (19)	0.000 (2)	−0.0015 (16)	−0.010 (2)
C16	0.0401 (18)	0.057 (3)	0.048 (2)	0.0007 (17)	−0.0030 (16)	−0.012 (2)
C17	0.0434 (19)	0.063 (3)	0.0382 (18)	−0.0067 (18)	0.0028 (15)	−0.0125 (19)
C18	0.127 (4)	0.097 (4)	0.045 (2)	0.009 (4)	0.014 (3)	−0.014 (3)
C19	0.066 (2)	0.063 (2)	0.062 (2)	0.007 (3)	0.001 (2)	−0.014 (2)
N1	0.0617 (17)	0.0490 (16)	0.0367 (14)	−0.0005 (19)	0.0051 (16)	−0.0077 (13)
O1	0.117 (3)	0.096 (3)	0.0341 (14)	0.021 (2)	−0.0022 (16)	−0.0001 (15)
O2	0.085 (2)	0.0635 (18)	0.0413 (13)	0.0139 (16)	−0.0039 (13)	−0.0106 (13)
O3	0.0662 (17)	0.086 (2)	0.0551 (15)	−0.0162 (16)	−0.0150 (13)	−0.0185 (16)
O4	0.0693 (17)	0.073 (2)	0.0410 (13)	0.0241 (15)	−0.0093 (13)	0.0071 (15)
O5	0.077 (2)	0.0691 (18)	0.0412 (14)	0.0187 (15)	0.0002 (13)	−0.0074 (13)
O6	0.087 (2)	0.078 (2)	0.0400 (14)	0.0153 (17)	−0.0033 (14)	−0.0105 (15)
S1	0.0499 (5)	0.0569 (6)	0.0334 (4)	0.0032 (5)	−0.0070 (4)	−0.0053 (4)
Br1	0.0684 (3)	0.0479 (2)	0.0752 (3)	0.0062 (2)	0.0019 (3)	−0.01215 (19)
O1W	0.089 (3)	0.072 (2)	0.0551 (19)	0.017 (2)	−0.0025 (18)	−0.0076 (19)

### Geometric parameters (Å, °)

**Table:**

C1—C2	1.377 (5)	C11—H11B	0.97
C1—C6	1.392 (6)	C12—C13	1.387 (6)
C1—C7	1.487 (5)	C12—C17	1.388 (6)
C2—C3	1.377 (5)	C13—C14	1.375 (6)
C2—H2	0.93	C13—H13	0.93
C3—C4	1.383 (5)	C14—C15	1.361 (6)
C3—S1	1.793 (3)	C14—H14	0.93
C4—C5	1.400 (5)	C15—O6	1.375 (5)
C4—Br1	1.893 (4)	C15—C16	1.395 (5)
C5—C6	1.381 (6)	C16—O5	1.366 (5)
C5—C8	1.510 (5)	C16—C17	1.372 (5)
C6—H6	0.93	C17—H17	0.93
C7—O1	1.210 (4)	C18—O6	1.406 (5)
C7—O2	1.293 (5)	C18—H18A	0.96
C8—H8A	0.96	C18—H18B	0.96
C8—H8B	0.96	C18—H18C	0.96
C8—H8C	0.96	C19—O5	1.417 (5)
C9—N1	1.451 (5)	C19—H19A	0.96
C9—H9A	0.96	C19—H19B	0.96
C9—H9B	0.96	C19—H19C	0.96
C9—H9C	0.96	N1—S1	1.607 (4)
C10—N1	1.472 (5)	O2—H2A	0.82
C10—C11	1.504 (6)	O3—S1	1.428 (3)
C10—H10A	0.97	O4—S1	1.432 (3)
C10—H10B	0.97	O1W—H2W	0.75 (3)
C11—C12	1.498 (6)	O1W—H1W	0.79 (3)
C11—H11A	0.97		
			
C2—C1—C6	120.0 (3)	H11A—C11—H11B	107.5
C2—C1—C7	120.1 (3)	C13—C12—C17	117.9 (4)
C6—C1—C7	119.9 (3)	C13—C12—C11	120.6 (4)
C3—C2—C1	119.9 (3)	C17—C12—C11	121.5 (4)
C3—C2—H2	120.1	C14—C13—C12	121.5 (4)
C1—C2—H2	120.1	C14—C13—H13	119.2
C2—C3—C4	120.0 (3)	C12—C13—H13	119.2
C2—C3—S1	115.4 (3)	C15—C14—C13	119.6 (4)
C4—C3—S1	124.6 (3)	C15—C14—H14	120.2
C3—C4—C5	121.3 (3)	C13—C14—H14	120.2
C3—C4—Br1	120.8 (3)	C14—C15—O6	124.7 (4)
C5—C4—Br1	117.9 (3)	C14—C15—C16	120.5 (4)
C6—C5—C4	117.6 (3)	O6—C15—C16	114.8 (4)
C6—C5—C8	120.4 (4)	O5—C16—C17	125.4 (3)
C4—C5—C8	122.0 (4)	O5—C16—C15	115.3 (3)
C5—C6—C1	121.3 (3)	C17—C16—C15	119.3 (4)
C5—C6—H6	119.3	C16—C17—C12	121.1 (4)
C1—C6—H6	119.3	C16—C17—H17	119.4
O1—C7—O2	123.0 (4)	C12—C17—H17	119.4
O1—C7—C1	123.6 (4)	O6—C18—H18A	109.5
O2—C7—C1	113.4 (3)	O6—C18—H18B	109.5
C5—C8—H8A	109.5	H18A—C18—H18B	109.5
C5—C8—H8B	109.5	O6—C18—H18C	109.5
H8A—C8—H8B	109.5	H18A—C18—H18C	109.5
C5—C8—H8C	109.5	H18B—C18—H18C	109.5
H8A—C8—H8C	109.5	O5—C19—H19A	109.5
H8B—C8—H8C	109.5	O5—C19—H19B	109.5
N1—C9—H9A	109.5	H19A—C19—H19B	109.5
N1—C9—H9B	109.5	O5—C19—H19C	109.5
H9A—C9—H9B	109.5	H19A—C19—H19C	109.5
N1—C9—H9C	109.5	H19B—C19—H19C	109.5
H9A—C9—H9C	109.5	C9—N1—C10	116.8 (3)
H9B—C9—H9C	109.5	C9—N1—S1	121.8 (3)
N1—C10—C11	113.0 (3)	C10—N1—S1	117.3 (2)
N1—C10—H10A	109.0	C7—O2—H2A	109.5
C11—C10—H10A	109.0	C16—O5—C19	117.7 (3)
N1—C10—H10B	109.0	C15—O6—C18	118.5 (4)
C11—C10—H10B	109.0	O3—S1—O4	118.65 (17)
H10A—C10—H10B	107.8	O3—S1—N1	108.82 (16)
C12—C11—C10	114.8 (3)	O4—S1—N1	109.40 (17)
C12—C11—H11A	108.6	O3—S1—C3	108.50 (17)
C10—C11—H11A	108.6	O4—S1—C3	105.31 (16)
C12—C11—H11B	108.6	N1—S1—C3	105.34 (15)
C10—C11—H11B	108.6	H2W—O1W—H1W	102 (6)
			
C6—C1—C2—C3	0.5 (5)	C13—C14—C15—C16	−0.3 (6)
C7—C1—C2—C3	179.9 (3)	C14—C15—C16—O5	179.5 (4)
C1—C2—C3—C4	0.5 (5)	O6—C15—C16—O5	−1.7 (5)
C1—C2—C3—S1	−178.5 (3)	C14—C15—C16—C17	0.0 (6)
C2—C3—C4—C5	−0.6 (5)	O6—C15—C16—C17	178.8 (3)
S1—C3—C4—C5	178.3 (3)	O5—C16—C17—C12	−179.9 (3)
C2—C3—C4—Br1	−179.8 (2)	C15—C16—C17—C12	−0.5 (5)
S1—C3—C4—Br1	−0.9 (4)	C13—C12—C17—C16	1.1 (5)
C3—C4—C5—C6	−0.2 (5)	C11—C12—C17—C16	−179.8 (3)
Br1—C4—C5—C6	179.0 (3)	C11—C10—N1—C9	65.3 (4)
C3—C4—C5—C8	−179.9 (4)	C11—C10—N1—S1	−136.7 (3)
Br1—C4—C5—C8	−0.7 (5)	C17—C16—O5—C19	2.0 (5)
C4—C5—C6—C1	1.2 (5)	C15—C16—O5—C19	−177.4 (3)
C8—C5—C6—C1	−179.1 (4)	C14—C15—O6—C18	0.3 (6)
C2—C1—C6—C5	−1.4 (5)	C16—C15—O6—C18	−178.5 (4)
C7—C1—C6—C5	179.2 (3)	C9—N1—S1—O3	−6.1 (4)
C2—C1—C7—O1	175.4 (4)	C10—N1—S1—O3	−163.0 (3)
C6—C1—C7—O1	−5.1 (6)	C9—N1—S1—O4	124.9 (3)
C2—C1—C7—O2	−3.0 (5)	C10—N1—S1—O4	−31.9 (3)
C6—C1—C7—O2	176.4 (3)	C9—N1—S1—C3	−122.3 (3)
N1—C10—C11—C12	66.2 (5)	C10—N1—S1—C3	80.9 (3)
C10—C11—C12—C13	68.6 (5)	C2—C3—S1—O3	129.1 (3)
C10—C11—C12—C17	−110.5 (4)	C4—C3—S1—O3	−49.9 (3)
C17—C12—C13—C14	−1.4 (6)	C2—C3—S1—O4	1.1 (3)
C11—C12—C13—C14	179.5 (4)	C4—C3—S1—O4	−177.9 (3)
C12—C13—C14—C15	1.0 (6)	C2—C3—S1—N1	−114.5 (3)
C13—C14—C15—O6	−178.9 (4)	C4—C3—S1—N1	66.5 (3)

### Hydrogen-bond geometry (Å, °)

**Table:**

*D*—H···*A*	*D*—H	H···*A*	*D*···*A*	*D*—H···*A*
O2—H2A···O1W	0.82	1.73	2.535 (4)	169
O1W—H1W···O1^i^	0.79 (3)	1.97 (4)	2.732 (5)	162 (6)
O1W—H2W···O5^ii^	0.75 (3)	2.46 (4)	3.066 (5)	139 (5)
O1W—H2W···O6^ii^	0.75 (3)	2.14 (4)	2.828 (5)	154 (5)

Symmetry codes: (i) −*x*+1, *y*−1/2, −*z*+1/2; (ii) *x*+1, *y*−1, *z*.
